# BCG Vaccination: A Role for Vitamin D?

**DOI:** 10.1371/journal.pone.0016709

**Published:** 2011-01-31

**Authors:** Maeve K. Lalor, Sian Floyd, Patricia Gorak-Stolinska, Rosemary E. Weir, Rose Blitz, Keith Branson, Paul E. Fine, Hazel M. Dockrell

**Affiliations:** 1 Department of Immunology and Infection, Faculty of Infectious and Tropical Diseases, London School of Hygiene & Tropical Medicine, London, United Kingdom; 2 Department of Infectious Disease Epidemiology, Faculty of Epidemiology and Public Health, London School of Hygiene & Tropical Medicine, London, United Kingdom; Institute of Infectious Diseases and Molecular Medicine, South Africa

## Abstract

**Background:**

BCG vaccination is administered in infancy in most countries with the aim of providing protection against tuberculosis. There is increasing interest in the role of vitamin D in immunity to tuberculosis. This study objective was to determine if there was an association between circulating 25(OH)D concentrations and BCG vaccination status and cytokine responses following BCG vaccination in infants.

**Methods:**

Blood samples were collected from UK infants who were vaccinated with BCG at 3 (n = 47) and 12 (n = 37) months post BCG vaccination. These two time-points are denoted as time-point 1 and time-point 2. Two blood samples were also collected from age-matched unvaccinated infants (n = 32 and 28 respectively), as a control group. Plasma vitamin D concentrations (25(OH)D) were measured by radio-immunoassay. The cytokine IFNγ was measured in supernatants from diluted whole blood stimulated with *M.tuberculosis* (*M.tb*) PPD for 6 days.

**Results:**

58% of infants had some level of hypovitaminosis (25(OH)D <30ng/ml) at time-point 1, and this increased to 97% 9 months later. BCG vaccinated infants were almost 6 times (CI: 1.8–18.6) more likely to have sufficient vitamin D concentrations than unvaccinated infants at time-point 1, and the association remained strong after controlling for season of blood collection, ethnic group and sex. Among vaccinees, there was also a strong inverse association between IFNγ response to *M.tb* PPD and vitamin D concentration, with infants with higher vitamin D concentrations having lower IFNγ responses.

**Conclusions:**

Vitamin D may play an immuno-regulatory role following BCG vaccination. The increased vitamin D concentrations in BCG vaccinated infants could have important implications: vitamin D may play a role in immunity induced by BCG vaccination and may contribute to non-specific effects observed following BCG vaccination.

## Introduction

BCG vaccination is administered in infancy in most countries with the aim of providing protection against mycobacterial infections such as tuberculosis and leprosy [1]. There has been debate about possible non-specific effects of BCG vaccination. Observational studies from low-income countries (Guinea-Bissau, Bangladesh, Papua New Guinea and Malawi) have indicated that BCG may have a beneficial effect on all-cause childhood mortality [2], [3], [4], [5], but there has been concern about possible confounding in these studies [6]. In one study, BCG was shown to influence responses to other vaccinations in infancy, with infants who receive BCG at birth along with their first Hepatitis B vaccine (HBV), having higher antibody, *in vitro* T cell proliferative and IFNγ responses to hepatitis B surface antigen two weeks after their HBV booster, than infants whose BCG vaccination was delayed [7]. As well as the evidence that BCG provides protection against mycobacterial infections such as tuberculosis and leprosy [8], studies have suggested that BCG vaccination may offer protection or beneficial effects in inflammatory and autoimmune conditions such as asthma, allergic diseases, Crohn's disease, diabetes, some cancers including bladder cancer [9] and helminth infections [10].

Vitamin D deficiency is now recognised as widespread [11] and is more common in TB patients than controls [12], [13], [14]. Patients with tuberculosis have, on average, lower serum concentrations of 25(OH)D than healthy controls [15]. A recent placebo controlled trial giving Vitamin D supplements along with TB chemotherapy failed to show evidence for an overall effect on clinical outcome or mortality, although this may have been due to the dose or dosing schedule [16].

In the last five years there has been renewed interest in the biological effects of Vitamin D on tuberculosis due to the growing evidence of the immunomodulatory properties of Vitamin D. Vitamin D alone has no direct anti-mycobacterial action, but its active metabolite 1α25(OH)_2_D modulates the host response to *M.tb* infection [17]. Toll Like Receptor (TLR) mediated activation of macrophages up-regulates expression of the Vitamin D receptor, leading to the induction of the antimicrobial peptide cathelicidin and to restriction of growth of *M.tb* [18]. The absence of adequate serum 25(OH)D (in African-American patients in the US) led to reduced induction of cathelicidin mRNA and impairment in mycobacterial growth inhibition [18]. Further studies confirmed that Vitamin D induced *M.tb* growth restriction is dependent on the induction of cathelicidin [19].

Vitamin D has also been shown to have an immuno-regulatory role *in vitro*. It has been shown to inhibit differentiation and maturation of dendritic cells and to act on T lymphocytes to inhibit T cell activation and proliferation resulting in altered cytokine expression [20]. It has recently been shown to be important in controlling T cell receptor signalling [21]. *In vitro* studies have shown that Vitamin D inhibits the generation of Th1 responses [22] and the production of IFNγ by previously activated T cells. It has been suggested that Vitamin D may promote the generation of regulatory T cells (Treg) [23]. Thus paradoxical effects of vitamin D have been observed in immunity of tuberculosis: vitamin D decreased Th1 mediated immunity, but increased bactericidal activity [24].

In this study, we measured 25(OH)D, which is considered to be the most reliable indicator of Vitamin D status [25], in plasma samples from UK infants who had received BCG vaccination and in age-matched infants who had not been vaccinated. We aimed to determine if there was an association between circulating 25(OH)D concentrations and BCG vaccination status and cytokine responses following BCG vaccination in UK infants.

## Materials and Methods

### Recruitment and study design

BCG vaccinated and unvaccinated infants were recruited in Redbridge and Waltham Forest Primary Care Trusts respectively after written informed consent from parents or guardians. These two similar, neighbouring Primary Care Trusts in North London had different BCG vaccination policies in place during the study period between March 2003 and November 2005; infants in Redbridge PCT were routinely offered BCG while those in Waltham Forest were not offered BCG. Two blood samples were taken, at time-points which we subsequently denote as time-point 1 and time-point 2. Blood samples were taken 3 months (infants aged approximately 6 months: n = 47 vaccinated, n = 32 unvaccinated) and 12 months (infants aged approximately 15 months: n = 37 vaccinated, n = 28 unvaccinated) post-vaccination in vaccinees, and controls were age-matched to the infants who received BCG vaccination. Diluted whole blood assays, IFNγ ELISAs and vitamin D assays were performed on blood samples at both time-points. Approval for the study was given by the Redbridge and Waltham Forest Health Authority Local Research Ethics Committee, and the Ethics Committee of the London School of Hygiene & Tropical Medicine.

### Vitamin D assay

Total plasma 25-hydroxyVitamin D (25(OH)D) was measured by Radio-immuno assay (RIA) with ^125^ I labelled 25(OH)D as a tracer, using a kit from Diasorin (Diasorin S.p.A., Saluggia, Italy) following the manufacturer's instructions. Briefly, 25(OH)D was extracted from plasma samples with acetonitrile. After centrifugation, an aliquot of the supernatant was incubated for 90 minutes with ^125^ I labelled 25(OH)D and 25(OH)D antibody. A second antibody precipitating complex was then added and incubated for 20 minutes, followed by the addition of a non-specific binding buffer for 20 minutes to reduce non-specific binding. After centrifugation, the supernatant was decanted and the radioactivity was measured on a γ-counter for two minutes or until 10,000 counts, whichever came first.

Hypovitaminosis was defined as plasma 25(OH)D <30 ng/ml (75 nmol/l), graded as severe Vitamin D deficiency (sVDD) for plasma 25(OH)D <10 ng/ml (25 nmol/l), mild Vitamin D deficiency (mVDD) for plasma 25(OH)D 10–19.9 ng/ml (25–50 nmol/l), and Vitamin D insufficiency (VDI) for plasma 25 (OH)D 20–29.9 ng/ml (50–75 nmol/ml) [12], [13].

The quality and reproducibility of the assay was determined using the quality controls provided with the kit. Two controls were provided and the coefficient of variation for all kits used was 17% for the low control and 18% for the high control. The controls fell within the acceptable range given by the manufacturer.

### Whole blood assay, IFNγ ELISA

Whole blood assays and ELISAs for IFNγ were carried out as described elsewhere [26], [27]. Heparinised whole blood was diluted 1 in 10 and cultured on the day of collection with the *M.tb* PPD (Statens Serum Institut, Copenhagen (SSI), RT49, lot 204) at a concentration of 5 µg/ml or medium alone (unstimulated) as the negative control. Cultures were incubated at 37°C with 5% CO_2_; supernatants were harvested on day 6 and stored at −70°C until assayed for IFNγ in single 100 µl samples by quantitative ELISA. Reproducibility was determined by duplicate positive control samples run on each ELISA plate, during the time-period of the study two different controls were used and the coefficient of variation between plates was 7% and 19% for the two controls.

### Statistical analysis

Data were analysed using Stata 10. Chi squared tests were used to assess 1) differences in Vitamin D concentrations between BCG vaccinated and unvaccinated infants; 2) differences in the proportion of IFNγ responders in BCG vaccinated and unvaccinated UK infants; and 3) differences in the proportion of IFNγ responders according to their Vitamin D response, among vaccinees. Linear regression was used to compare mean differences in Vitamin D concentrations, and logistic regression to calculate odds ratios for Vitamin D sufficiency, according to BCG vaccination status, season of blood collection, ethnic group and sex. Ethnic group was divided into “Caucasian” (which included infants whose mothers described them on the consent form as “White British, White Irish or White other”) and “Non-Caucasian” (which included “Asian Pakistani, Black African, Black Caribbean, Mixed White and Black Caribbean, Mixed White and Black African, Mixed White and Asian”). Stratified analyses of the effect of BCG vaccination on the vitamin D response, by season of blood collection, ethnic group and sex, were conducted to investigate confounding and effect modification. Finally, multivariable linear and logistic regression analysis was conducted to calculate mean differences and odds ratios for the effect of BCG vaccination on the vitamin D concentration, controlled for other infant characteristics. Multivariable linear regression was used to estimate the effect of vitamin D concentration on the IFNγ response, controlled for season of birth and season of blood collection.

## Results

### Vitamin D sufficiency

**Figure 1:**
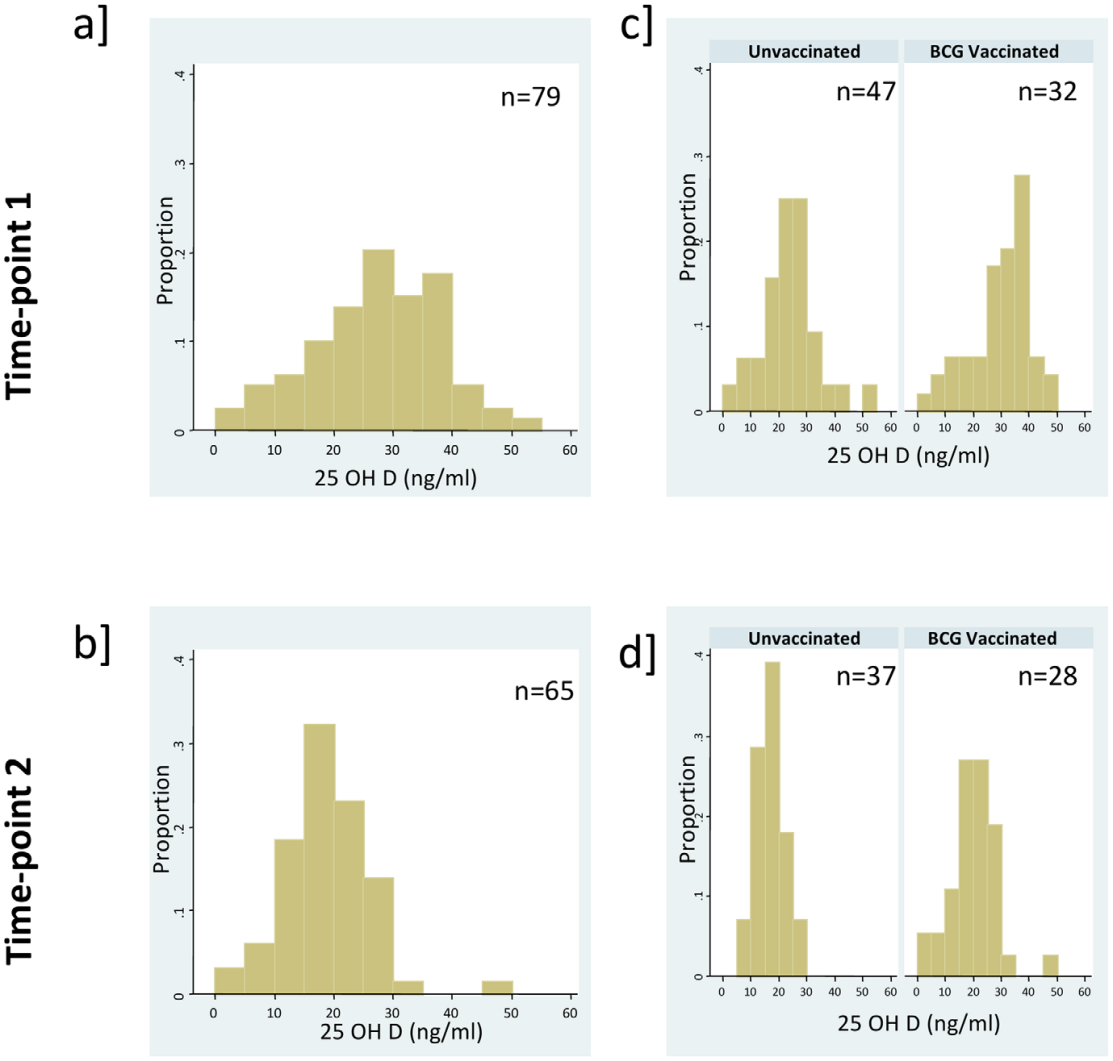
Histograms of 25(OH)D at time-point 1 and 2. 25(OH)D was measured in plasma samples from infants 3 and 12 months post BCG vaccination, and in age-matched unvaccinated controls. 25(OH)D in BCG vaccinated and unvaccinated infants **a**] in vaccinated infants and unvaccinated controls combined at time-point 1 and **b**] in vaccinated infants and unvaccinated controls combined at time-point 2; 25(OH)D in infants grouped according to BCG vaccination status at **c**] time-point 1 and **d**] time-point 2.

Vitamin D (25(OH)D) was measured in plasma samples from infants 3 months post BCG (n = 47) and in unvaccinated controls (n = 32) (n = 79 total) and in samples from infants 12 months post BCG (n = 37) and in age-matched unvaccinated controls (n = 28) (n = 65 total). The median concentration of Vitamin D in all the UK infants measured at time-point 1 was 28.7 ng/ml, which decreased significantly by time-point 2 when the median was 18.9 ng/ml (P<0.0001) (**Figure 1**)**.**

At time-point 1, only 42% (33/79) of infants were considered to have Vitamin D sufficiency (VDS) (>30 ng/ml), while the remaining infants had some level of hypovitaminosis (<30 ng/ml): 8% (6/79) had severe Vitamin D deficiency (sVDD) (0–9.9 ng/ml), 17% (13/79) had mild Vitamin D deficiency (mVDD) (10–19.9 ng/ml) and 34% (27/79) had Vitamin D insufficiency (VDI) (20–29.9 ng/ml). Nine months later, at time-point 2, only 3% (2/65) of all infants had Vitamin D sufficiency, while the remaining infants showed evidence of hypovitaminosis: 9% (6/65) sVDD, 51% (33/65) mVDD and 37% (24/65) VDI (**Tables 1** **and** **2**).

**Table 1:** Infant vitamin D status at time-point 1, by infant characteristics, with groups compared according to mean differences and crude odds ratios for Vitamin D Sufficiency.

Exposure		No. of infants	No. infants (%) with sVDD * (<10 ng/ml)	No. infants (%) with mVDD * (10–19.9 ng/ml)	No. infants (%) with VDI * (20–29.9 ng/ml)	No. infants (%) with VDS * (>30 ng/ml)	Mean Vitamin D concentration (ng/ml)	Mean difference (95% CI)	P value (linear regression)	Infants with VDS (>30 ng/ml)	
										Crude Odds ratio (95% CI)	P value (LR Test) **
All infants		79	6 (7.6)	13 (16.5)	27 (34.2)	33 (41.7)	27.6				
BCG Vaccination status	Unvaccinated	32	3 (9.4)	7 (21.9)	16 (50.0)	6 (18.8)	23.9		0.01	1	0.0007
	BCG Vaccinated	47	3 (9.4)	6 (12.8)	11 (23.4)	27 (57.5)	30.2	6.3 (1.5,11.1)		5.85 (1.84, 18.57)	
Season of blood collection	Autumn	23	1 (4.4)	4 (17.4)	4 (17.4)	14 (60.9)	29.9	8.6 (2.5, 14.8)	0.007	8.81 (1.99, 38.95)	0.011
	Winter	12	3 (25.0)	0 (0.0)	5 (41.7)	4 (33.3)	25.2	4 (−3.4, 11.3)	0.287	2.83 (0.51, 15.77)	
	Spring	20	2 (10.0)	8 (40.0)	7 (35.0)	3 (15.0)	21.2			1	
	Summer	24	0 (0.0)	1 (4.2)	11 (45.8)	12 (50.0)	32.0	10.8 (4.7, 16.9)	0.001	5.67 (1.31, 24.53)	
Ethnic group ***	Caucasian	59	6 (10.2)	9 (15.3)	20 (33.9)	24 (40.7)	27.0		0.41	1	0.61
	Not Caucasian	19	0 (0.0)	4 (21.1)	6 (31.6)	9 (47.4)	29.4	2.4 (−3.4, 8.1)		1.3 (0.46, 3.74)	
Sex	Female	38	4 (10.5)	5 (13.2)	11 (29.0)	18 (47.4)	27.9	0.5 (−4.3, 5.4)	0.83	1.56 (0.63, 3.88)	0.33
	Male	41	2 (4.9)	8 (19.5)	16 (39.0)	15 (36.6)	27.4			1	

*severe Vitamin D Deficiency (sVDD), mild Vitamin D Deficiency (mVDD), Vitamin D Insufficiency (VDI) and Vitamin D Sufficiency (VDS).

**LR =  likelihood ratio test.

***1 data point missing as ethnic group “not stated”.

**Table 2:** Infants vitamin D status at time-point 2 by infant characteristics, with groups compared according to mean difference and crude odds ratios for no vitamin D deficiency (>20 ng/ml).

Exposure		No. of infants	No. infants (%) with sVDD * (<10 ng/ml)	No. infants (%) with mVDD * (10–19.9 ng/ml)	No. infants (%) with VDI * (20–29.9 ng/ml)	No. infants (%) with VDS * (>30 ng/ml)	No. infants (%) without deficiency (>20 ng/ml)	Mean Vitamin D (ng/ml)	Mean difference (95% CI)	P value (regression)	Infants without deficiency (>20 ng/ml)	
											Crude Odds ratio (95% CI)	P value (LR Test) **
All infants		65	6 (9.2)	33 (50.8)	24 (36.9)	2 (3.1)	26 (40.0)	18.8				
BCG Vaccination status	Unvaccinated	28	2 (7.1)	19 (67.9)	7 (25.0)	0 (0.0)	7 (25.0)	16.8		0.057	1	0.033
	BCG Vaccinated	37	4 (10.8)	14 (37.8)	17 (46.0)	2 (5.4)	19 (51.4)	20.3	3.5 (−0.1, 7.0)		3.17 (1.03, 9.70)	
Season of blood collection	Autumn	12	1 (8.3)	3 (25.0)	8 (66.7)	0 (0.0)	8 (66.7)	20.7	4.7 (−0.5, 9.4)	0.052	10 (1.44, 69.20)	0.0051
	Winter	12	1 (8.3)	9 (75.0)	1 (8.3)	1 (8.3)	2 (16.7)	18.2	2.2 (2.5, 6.9)	0.36	1	
	Spring	28	4 (14.3)	17 (60.7)	7 (25.0)	0 (0.0)	7 (25.0)	16.0			1.67 (0.29, 9.52)	
	Summer	12	0 (0.0)	4 (33.3)	7 (58.3)	1 (8.3)	8 (66.7)	23.2	7.2 (2.5, 12.0)	0.003	10 (1.44, 69.20)	
Ethnic group***	Caucasian	50	2 (4.0)	27 (54.0)	19 (38.0)	2 (4.0)	21 (42.0)	19.5	3.3 (−1.0, 7.5)	0.13	1.45 (0.43, 4.93)	0.55
	Not Caucasian	15	4 (26.7)	6 (40.0)	5 (33.3)	0 (0.0)	5 (33.3)	16.3			1	
Sex	Female	33	0 (0.0)	20 (60.6)	12 (36.4)	1 (3.0)	13 (39.4)	20.0	2.5 (−1.1, 6.1)	0.17	0.95 (0.35, 2.58)	0.92
	Male	32	6 (18.8)	13 (40.6)	12 (37.5)	1 (3.1)	13 (40.6)	17.5			1	

Severe Vitamin D Deficiency (sVDD), mild Vitamin D Deficiency (mVDD), Vitamin D Insufficiency (VDI) and Vitamin D Sufficiency (VDS).

**LR =  likelihood ratio test.

***1 data point missing as ethnic group “not stated”.

### Factors associated with Vitamin D sufficiency

There was no evidence that Vitamin D concentration was affected by ethnicity or sex at time-point 1, while there was strong evidence that BCG vaccination status had an effect on Vitamin D levels. BCG vaccinated infants' mean Vitamin D concentrations were 6.3 ng/ml higher than unvaccinated infants (P = 0.01) and the odds of a vaccinated infant having sufficient Vitamin D were 5.85 times higher than an unvaccinated infant (C.I 1.84, 18.57, P = 0.0007) (**Figure 1** **,** **Table 1**). There was also evidence that season of blood sample collection had an effect on the concentration of Vitamin D, with blood samples which were taken in spring and winter having lower Vitamin D than those taken in summer and autumn (P = 0.011).

By time-point 2, Vitamin D levels had decreased significantly (P<0.0001), and only 3% (2/65) of infants had “sufficient” Vitamin D according to the defined cut-offs used. In order to assess associations with Vitamin D status infants were divided into those who had less than 20 ng/ml (with severe or mild deficiency) and those with greater than 20 ng/ml (with insufficiency or sufficiency). Again, there was evidence that Vitamin D concentrations were higher among those who were BCG vaccinated compared to those who had not received BCG vaccination, although this association was weaker than at 3 months post BCG. BCG vaccinated infants had a mean Vitamin D concentration that was 3.5 ng/ml higher than unvaccinated infants and BCG vaccinated infants had 3.2 times the odds of having Vitamin D levels above 20 ng/ml than unvaccinated infants (P = 0.033) (**Table 2**). There was also strong evidence of an association with season of blood sample collection, with blood samples taken in the summer and autumn having higher Vitamin D levels (P = 0.0051). There was no evidence of an association between vitamin D concentration and either ethnic group or sex, although there was a trend of higher Vitamin D concentrations in Caucasian infants compared to non-Caucasians and in females compared to males (P = 0.13, P = 0.17 respectively) (**Table 2**).

Vitamin D levels remained associated with BCG vaccination status at time-point 1, both after controlling one at a time for season of blood collection (P = 0.004), ethnic group (P = 0.0005), and sex (P = 0.0008), and after controlling simultaneously for these factors (P = 0.006), although the association was weakened (mean difference 6.3 unadjusted, 3.3 after controlling for all of season of blood collection, sex, and ethnic group) (**Table 3**). Restricting to Caucasian infants to remove any residual confounding by ethnicity (n = 59) confirmed the association remained strong; the BCG vaccinated group were 7.2 times more likely to be vitamin D sufficient, than the unvaccinated group. By time-point 2, Vitamin D levels remained associated with BCG vaccination status, and controlling for season of blood collection, ethnic group and sex strengthened the association slightly (mean difference 3.5 unadjusted, 4.4 in adjusted analysis) (**Table 4**).

**Table 3:** Infant vitamin D concentrations at time-point 1, 3 months post-BCG vaccination compared to age-matched controls: mean differences and odds ratios for the effect of BCG vaccination, stratified on and controlled for season of blood collection, ethnic group and sex.

		Unvaccinated mean (sd)	BCG vaccinated mean (sd)	Mean difference between vac and unvac, controlling for other infants characteristics	No. unvac infants (%) with Vitamin D >30 ng/ml	No. BCG vac infants (%) with Vitamin D >30 ng/ml	Odds ratio	95% CI	P value
**Overall**		23.9 (10.1)	30.2 (10.7)	6.3 (1.5,11.1) *	6/32 (18.8)	27/47 (57.5)	5.85 *	1.84, 18.57	<0.001
**Stratified on season of blood collection**	**Autumn**	30.3 (13.1)	29.6 (11.4)	3.7 (−1.3,8.6) **	4/9 (44.4)	10/14 (71.4)	5.54 **	1.49, 20.66	0.004
	**Winter**	23.5 (8.7)	26.8 (17.2)		1/6 (16.7)	3/6 (50.0)			
	**Spring**	18.7 (7.4)	25.9 (10.8)		1/13 (7.7)	2/7 (28.6)			
	**Summer**	26.8 (2.4)	33.1 (7.3)		0/4 (0.0)	12/20 (60.0)			
**Stratified on ethnic group**	**Caucasian**	23.4 (10.5)	30.6(11.8)	6.3 (1.2,11.3) **	5/29 (17.2)	19/30 (63.3)	6.33 **	1.92, 20.84	0.0005
	**Not caucasian**	28.8 (1.6)	29.5 (8.9)		1/3 (33.3)	8/16 (50.0)			
**Stratified on sex**	**Female**	23.0 (12.2)	30.7 (11.5)	6.3 (1.5,11.1) **	3/14 (21.4)	15/24 (62.5)	5.76 **	1.80, 18.45	0.0008
	**Male**	24.5 (8.4)	29.6 (10.0)		3/18 (16.7)	12/23 (52.2)			
**Adjusted for season of blood collection, ethnic group and sex**				3.3 (−2.0,8.7) **			5.86 **	1.64, 20.92	0.006

*unadjusted (“crude”),

**adjusted.

**Table 4:** Infant vitamin D concentrations at time-point 2, 12 months post-BCG vaccination compared to age-matched controls: mean differences and odds ratios for the effect of BCG vaccination, stratified on and controlled for season of blood collection, ethnic group and sex.

		Unvaccinated mean (sd)	BCG vaccinated mean (sd)	Mean difference between vac and unvac, controlling for other infants characteristics	No. unvac infants (%) with Vitamin D >20 ng/ml	No. BCG vac infants (%) with Vitamin D >20 ng/ml	Odds ratio	95% CI	P value
**Overall**		16.8 (5.6)	20.3 (8.1)	3.5 (−0.1, 7.0) *	7/28 (25.0)	19/37 (51.4)	3.17 *	1.1, 9.2	0.035
**Stratified on season of blood collection**	**Autumn**	20.7 (6.6)	20.6 (6.3)	2.9 (−0.7, 6.6) **	4/6 (66.7)	4/6 (66.7)	3.49 **	0.9, 13.0	0.063
	**Winter**	14.5 (5.2)	23.4 (14.3)		0/7 (0.0)	2/5 (40.0)			
	**Spring**	15.6 (3.5)	16.3 (7.2)		2/13 (15.4)	5/15 (33.3)			
	**Summer**	14 (nd)	24.1 (4.5)		0/1 (0.0)	8/11 (72.7)			
**Stratified on ethnic group**	**Caucasian**	17.3 (5.3)	22.0 (8.3)	4.9 (1.3, 8.6) **	7/26 (26.9)	14/24 (58.3)	4.17 **	1.3, 13.2	0.016
	**Not caucasian**	10.9 (7.1)	17.1 (7.1)		0/2 (0.0)	5/13 (38.5)			
**Stratified on sex**	**Female**	17.8 (6.3)	21.4 (7.7)	3.3 (−0.3, 6.9) **	3/13 (23.1)	10/20 (50.0)	3.21 **	1.1, 9.4	0.034
	**Male**	15.9 (4.9)	19.0 (8.7)		4/15 (26.7)	9/17 (52.9)			
**Adjusted for season of blood collection, ethnic group and sex**				4.4 (0.4, 8.3) **			6.1 **	1.3, 28.3	0.021

*unadjusted (“crude”),

**adjusted, nd is not done as only 1 infant in group.

### Association of Vitamin D and IFNγ responses to *M.tb* PPD

**Figure 2:**
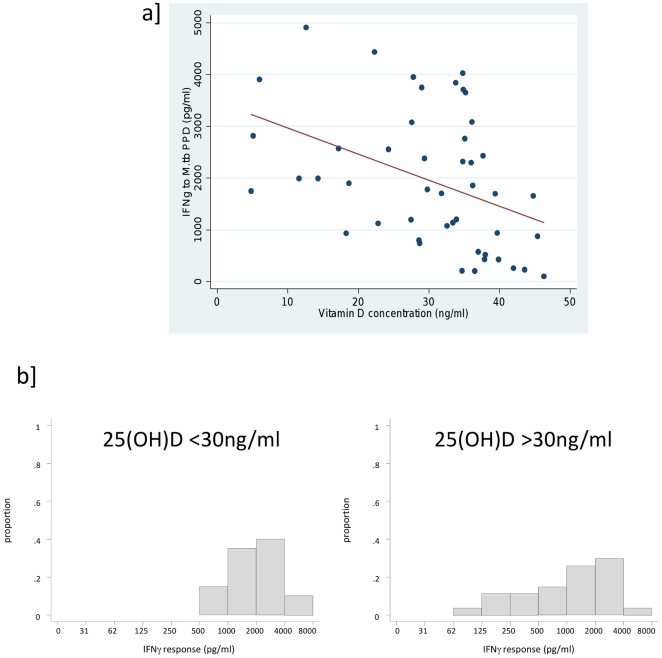
Association of IFNγ to *M.tb* PPD with 25(OH)D concentrations 3 months post BCG vaccination. 25 (OH) D was measured in plasma samples and IFNγ to *M.tb* PPD was measured by ELISA from supernatants from 6 day cultures of diluted whole blood from infants 3 months post BCG vaccination. **a**] Dot plot where individuals are represented by dots, and lines represents linear fit of responses **b**] Histograms of IFNγ response to *M.tb* PPD response in infants with hypovitaminosis (<30 ng/ml) and in infants with sufficient 25(OH)D.

At 3 months post BCG vaccination, there was strong evidence of an inverse association among vaccinees between IFNγ response to *M.tb* PPD and Vitamin D concentrations, with a moderately strong negative correlation (r = −0.4, P = 0.004) (**Figure 2** **,** **Table 3**). BCG vaccinated infants with higher levels of Vitamin D (>30 ng/ml) had lower mean IFNγ responses to *M.tb* PPD than infants with lower Vitamin D levels 3 months post BCG (P = 0.030). There was also a trend of an inverse association at 12 months, though this did not reach statistical significance and the confidence interval was wide (P = 0.255) (**Table 5**). After controlling for season of blood collection, the mean difference in IFNγ responses in infants with low and high Vitamin D increased slightly (**Table 5**).

**Table 5:** Mean IFNγ response to *M.tb* PPD by vitamin D status, controlling for season of blood collection at 3 and 12 months post BCG, among vaccines.

		No. of infants	Mean IFNγ to *M.tb* PPD (pg/ml)	Mean difference in IFNγ to *M.tb* PPD between infants with low and high vitamin D (95% CI)	P value
3 months post BCG	Hypovitaminosis (<30 ng/ml)	20	2426	829 (84, 1575) *	0.030
	Sufficient Vitamin D (>30 ng/ml)	27	1597		
	Adjusted for season of blood collection			883 (83, 1683) **	0.031
12 months post BCG	Vitamin D deficiency (<20 ng/ml)	18	1542	466 (−352, 1283) *	0.255
	Vitamin D (>20 ng/ml)	19	1076		
	Adjusted for season of blood collection			523 (−375,1420) **	0.244

*unadjusted (“crude”),

**adjusted.

## Discussion

### Vitamin D Sufficiency

A high percentage (58% at age 6 months and 97% at age 15 months) of Vitamin D hypovitaminosis (25(OH)D <30 ng/ml) was found in this study of UK infants. Globally, it has been recognised that there is widespread Vitamin D deficiency [28], however there are no recently published data on the prevalence of Vitamin D deficiency in UK children aged <18 months. A study examining Vitamin D levels in children in London with active or latent tuberculosis recently showed that 86% (55/64) of children (aged 0.1 to 17 years) had hypovitaminosis, most of whom were South Asian or Black African [29]. Much of the focus of research in the UK has been on Vitamin D deficiency in Asian children [30] however, Vitamin D insufficiency has been shown to be common in pregnant Caucasian women from Southampton [31], resulting in predictions that Vitamin D insufficiency is also common in Caucasian infants in the UK, given that a woman's Vitamin D serum concentration during pregnancy is strongly predictive of her child's concentration at birth [25]. The results from our study provided no evidence of an effect of ethnicity on vitamin D concentrations in UK infants, as similar levels of hypovitaminosis were seen in Caucasian and non-Caucasian infants.

Our results provide evidence for a decrease in Vitamin D levels in UK infants between age 6 and 15 months. One explanation for the observed decrease in Vitamin D levels could be due to higher levels shortly after birth due to uptake via the placenta, followed by a decrease over time if infants were continuously breast fed (without supplementation, breast milk has been shown to contain low levels of Vitamin D [25]).

### Plasma vitamin D is associated with BCG vaccination status

Many factors have been found to influence circulating Vitamin D levels such as sunlight exposure, skin pigmentation, use of sunscreen, latitude, season, clothing, diet and supplementation [25]. Our results are the first to show that BCG vaccination influences Vitamin D concentrations. At time-point 1, the odds of having sufficient Vitamin D levels were approximately six times higher in vaccinated than unvaccinated infants, and this effect remained strong after controlling for seasonal effects. There have been studies which have shown effects of BCG on asthma, allergic diseases, cancers, and immune responses to other childhood vaccinations [7], [9]. These effects of BCG vaccination could be partly due to the vaccine's effect in activating antigen presenting cells such as dendritic cells and macrophages, with resulting potent Th1 activation. Such effects may be particularly important in infants, since they have immature dendritic cells at birth that may be matured following BCG vaccination [32]. There is increasing, although inconsistent, evidence that BCG vaccination plays a role in the maturation of the immune system and in the development and balance of regulatory pathways [9]. Vitamin D has also been shown to play a role in immuno-regulation, and is thought to be involved in many autoimmune and inflammatory diseases such as multiple sclerosis, type-1 diabetes, rheumatoid arthritis and systemic lupus erythematosis; cardiovascular diseases and cancers [20]. Given the finding that BCG vaccinated infants had higher concentrations of plasma Vitamin D than unvaccinated infants, it is possible that part of the non-specific effects that have been attributed to BCG vaccination may be due to increased Vitamin D levels. It is still unclear what the ideal magnitude of circulating 25(OH)D is, and thus it is not known whether the difference in magnitude of 25(OH)D between vaccinated and unvaccinated infants seen in this study is clinically significant.

### Proposed mechanism of BCG vaccination resulting in increased vitamin D

BCG vaccination is given intradermally, where it is first phagocytosed by macrophages and antigens are then presented and processed by macrophages and dendritic cells. It is probable that this is followed by trafficking to the lymph nodes in mobilised dendritic cells, and that T cell responses are then stimulated in the lymph nodes. In the last 5 years, there has been much evidence that vitamin D synthesis occurs not just by the classical pathway in the liver and kidneys, but that it can also occur within macrophages and dendritic cells as they possess all the necessary enzymes for its synthesis [18], [20], [33], [34], [35]. *M.tb* antigens activate TLR1 and TLR2 on macrophages resulting in increased production of 1α-hydroxylase and increased expression of VDR resulting in the production of cathelicidin, which has antimycobacterial properties [18]. We propose that mycobacterial antigens in BCG may also increase production of the 25 hydroxylase enzymes, resulting in increased production of 25(OH)D.

The scar generated following BCG vaccination matures and heals over several months, suggesting that immunological processes occur at the site of inoculation for some time. The increased vitamin D concentrations observed in this study could be due to increased local production in macrophages and dendritic cells following BCG vaccination. It is, however, surprising that this should result in sustained raised plasma concentrations of vitamin D, suggesting that the increased vitamin D production is not just local to the BCG inoculation. It is possible that the local increased production of 1,25(OH)_2_D_3_ plays a role by influencing the migration of the activated DCs from the site of vaccination to the lymph nodes [36], [37] resulting in further production of vitamin D there.

### Vitamin D association with IFNγ response to *M.tb* PPD

Vitamin D has been shown to play an immuno-regulatory role [20], [23], and in particular has been shown to regulate IFNγ production [38], [39]. It is possible that Vitamin D is produced as part of the immuno-regulatory mechanism to control the pro-inflammatory response produced following vaccination. Our results show that 25(OH)D concentration is inversely associated with the IFNγ response to *M.tb* PPD following BCG vaccination 3 months post vaccination, but that this effect is weaker at 12 months. The weakening of the effect at 12 months could be due to lower levels of Vitamin D being present at 12 compared to 3 months.

The paradoxical effects of vitamin D on Th1 mediated immunity have been observed in tuberculosis whereby Vitamin D treatment is associated with decreased Th1 mediated immunity, but increased bactericidal activity [24]. Vitamin D supplementation of participants in a study resulted in increased killing of BCG *in vitro* [40]. It would be interesting to further explore whether the increased levels of Vitamin D observed in BCG vaccinated infants are beneficial or harmful for the long term protection BCG offers against tuberculosis and if the rate of tuberculosis is different according to Vitamin D status, among vaccinees. Increased killing of BCG may not be beneficial if BCG needs to remain alive within the body to maintain protection against tuberculosis. On the other hand, perhaps part of the mechanism of action of BCG against tuberculosis is through increased production of Vitamin D resulting in cathelicidin production and killing of *M.tb*. Given that the Th1 response is protective, but also causes pathology, Vitamin D may provide the ideal response: capable of inducing increased bactericidal activity coupled with a decreased, but present, Th1 response. Vitamin D status may thus have potential as an additional biomarker of protection.

This study has the limitation that it was a small study and the information on several known factors that influence Vitamin D concentrations in infants, namely infants' feeding practices, vitamin supplementation in mothers or infants, and exposure to sunlight, were not collected. However, it is unlikely that these factors were associated with child vaccination status, as whether or not infants received BCG vaccination depended solely on which one of two neighbouring Primary Care Trusts they resided in. It is thus unlikely that these known “risk factors” have confounded the observed associations between BCG vaccination status and Vitamin D concentrations. It is policy in both Primary Care Trusts to follow Department of Health Guidelines on vitamin D supplementation, and supplementation should therefore have been similar in both settings during this study.

We believe that the findings of this observational study are both interesting and important. Further studies, including a randomised controlled trial, are now required to confirm that differences between vaccinated and unvaccinated infants were not due to differences in supplementation between the two sites. These studies could be conducted in the UK where many infants are not BCG vaccinated, in an area where BCG is not routinely offered and in the absence of vitamin D supplementation, by randomising infants to receive BCG vaccination or not. The findings of our study, if confirmed in a randomised trial, would have important implications for BCG vaccination and Vitamin D supplementation policies.
